# Three-dimensional self-gated cardiac MR imaging for the evaluation of myocardial infarction in mouse model on a 3T clinical MR system

**DOI:** 10.1371/journal.pone.0189286

**Published:** 2017-12-07

**Authors:** Xiaoyong Zhang, Bensheng Qiu, Zijun Wei, Fei Yan, Caiyun Shi, Shi Su, Xin Liu, Jim X. Ji, Guoxi Xie

**Affiliations:** 1 Centers for Biomedical Engineering, University of Science and Technology of China, Hefei, China; 2 Paul C. Lauterbur Research Center for Biomedical Imaging, Institute of Biomedical and Health Engineering, Shenzhen Institutes of Advanced Technology, Chinese Academy of Sciences, Shenzhen, China; 3 Department of Electrical and Computer Engineering, Texas A&M University, College Station, Texas, United States of America; 4 The Sixth Affiliated Hospital of Guangzhou Medical University, Guangzhou, China; 5 Department of Biomedical Engineering, Guangzhou Medical University, Guangzhou, China; University of California San Francisco, UNITED STATES

## Abstract

**Purpose:**

To develop and assess a three-dimensional (3D) self-gated technique for the evaluation of myocardial infarction (MI) in mouse model without the use of external electrocardiogram (ECG) trigger and respiratory motion sensor on a 3T clinical MR system.

**Methods:**

A 3D T1-weighted GRE sequence with stack-of-stars sampling trajectories was developed and performed on six mice with MIs that were injected with a gadolinium-based contrast agent at a 3T clinical MR system. Respiratory and cardiac self-gating signals were derived from the Cartesian mapping of the k-space center along the partition encoding direction by bandpass filtering in image domain. The data were then realigned according to the predetermined self-gating signals for the following image reconstruction. In order to accelerate the data acquisition, image reconstruction was based on compressed sensing (CS) theory by exploiting temporal sparsity of the reconstructed images. In addition, images were also reconstructed from the same realigned data by conventional regridding method for demonstrating the advantageous of the proposed reconstruction method. Furthermore, the accuracy of detecting MI by the proposed method was assessed using histological analysis as the standard reference. Linear regression and Bland-Altman analysis were used to assess the agreement between the proposed method and the histological analysis.

**Results:**

Compared to the conventional regridding method, the proposed CS method reconstructed images with much less streaking artifact, as well as a better contrast-to-noise ratio (CNR) between the blood and myocardium (4.1 ± 2.1 vs. 2.9 ± 1.1, *p* = 0.031). Linear regression and Bland-Altman analysis demonstrated that excellent correlation was obtained between infarct sizes derived from the proposed method and histology analysis.

**Conclusion:**

A 3D T1-weighted self-gating technique for mouse cardiac imaging was developed, which has potential for accurately evaluating MIs in mice at 3T clinical MR system without the use of external ECG trigger and respiratory motion sensor.

## Introduction

Recently, many kinds of animal models have become useful tools for the study of human cardiac disease processes. In particular, due to the genetic similarity with humans and the relatively low cost of maintenance, the mouse has evolved as a powerful animal model [1, 2]. As a result, many different imaging techniques, such as cardiac magnetic resonance (CMR) imaging [3–5], computed tomography (CT) [6], and echocardiography [7], are being developed to analyze the mouse model for the study of cardiovascular pathophysiological mechanisms and the evaluation of novel therapies of human disease. Among these techniques, CMR has been identified as an accurate modality to assess myocardial infarction (MI) in mice, because of its noninvasive nature and its capacity for high temporal and spatial resolution cardiac imaging [8, 9].

Routine CMR techniques for assessing MI in mouse are mainly based on external electrocardiogram (ECG) trigger and respiratory motion sensor to eliminate the cardiac and respiratory motion artifacts [10]. These techniques with external ECG and respiratory motion sensor can realize two dimensional (2D) or three dimensional (3D) cardiac imaging, which have been approved as promising tools for accurately assessing MI in mouse [11–13]. However, there remain some technical challenges on using the external ECG and respiratory motion sensor. First, manipulations of ECG and respiratory motion sensor are cumbersome because the size of mouse is rather small and the mouse heartbeat and respiratory rates are rapid (heart rate, 400–550 beats per minute, and respiratory rate, 60–150 breaths per minute) [14]. Second, the damage of myocardium associated with MI may result in cardiac arrhythmia, which can lead to irregular heartbeats and thus incorrect ECG triggering, resulting in cardiac motion artifacts [15]. Third, ECG is prone to produce unreliable triggers caused by interferences with the scanner’s magnetic and RF fields, which also results in cardiac motion artifacts [16, 17]. Thus, CMR techniques for detecting MI in mice without external ECG and respiratory motion sensor would be valuable and highly desired for MI study.

Self-gated CMR technique has potential to be an alternative tool for mouse cardiac imaging without the use of external ECG and respiratory motion sensor [18]. The technique extracts cardiac and respiratory motion signals from the self-gating data and subsequently realigns the acquired data to eliminate the cardiac and respiratory motion artifacts. The technique can achieve 2D or 3D CMR imaging and have been shown to be a valuable tool for cardiac MR imaging in mouse [19–23]. However, the difficulties associated with imaging MI in mouse model are related to basic animal physiological characteristics. For example, to observe the very small size of MI in the mouse model, 3D high spatial resolution imaging is required. which requires a long MR scan to acquire enough data for ensuring accurate and artifact-free reconstruction of cardiac images [23]. However, a long MR scan may be difficult when studying a fragile animal that has high physiological/metabolic stability demands throughout the experiment [23]. Thus, accelerating self-gated 3D cardiac imaging in mouse model of MI is therefore of interest.

A useful strategy for accelerating data acquisition in MR imaging is to exploit the spatial or/and temporal sparsity based on the compressed sensing (CS) theory. However, previous studies only achieve 2D real-time cardiac imaging for mouse heart [24, 25]. Compared to 2D cardiac imaging, 3D imaging can provide a better description of the disease models because there is no slice gap and has much higher spatial resolution along with the partition direction [23]. In this study, we present a novel rapid 3D self-gated CMR method for assessing MI in mouse. The method employs a 3D T1-weighted gradient echo (GRE) sequence with stack-of-stars sampling trajectories for data acquisition and uses the advantages of compressed sensing for image reconstruction. Our preliminary *in vivo* study demonstrated that the proposed approach can accurately detect MI in a 1.5 min scan, obviating the need for external ECG and respiratory motion sensor.

## Materials & methods

### Ethics statement

All experimental procedures were approved by the Animal Ethics Committee and conducted with strict adherence to the guidelines published by the Shenzhen Institute of Advanced Technology, Chinese Academy of Sciences (Permit Number: SIAT-IRB-150213-YGS-ZHR-YF-A0094-3). All surgeries were performed under sodium pentobarbital anesthesia, and all efforts were made to minimize suffering.

### Animal preparation

Six C57BL/6J male mice were purchased from the Guangdong Medical Animal Experiment Center and all mice were housed in the local pathogen-free environment before experiments. Briefly, each mouse was anesthetized with sodium pentobarbital (50 mg/kg body weight) by intraperitoneal injection and maintained at 37°C. The anaesthetized mouse was intubated endotracheally in a supine position and placed on a rodent ventilator (Chengdu Taimeng Software Co., LTD, Chengdu, China). Regional myocardial ischemia was induced by transient ligation of the left anterior descending coronary artery (LAD) using a 5.0 Protune suture. Ligation was confirmed by observation of ST-elevation in a three-lead ECG and removed after 30 minutes to allow reperfusion. Finally, the chest and skin of the conducted mouse was closed with a 4.0 Protune suture and air was evacuated from the chest cavity. In addition, penicillin was injected intraperitoneally to avoid infection.

### Sequence and data acquisition

**Fig 1:**
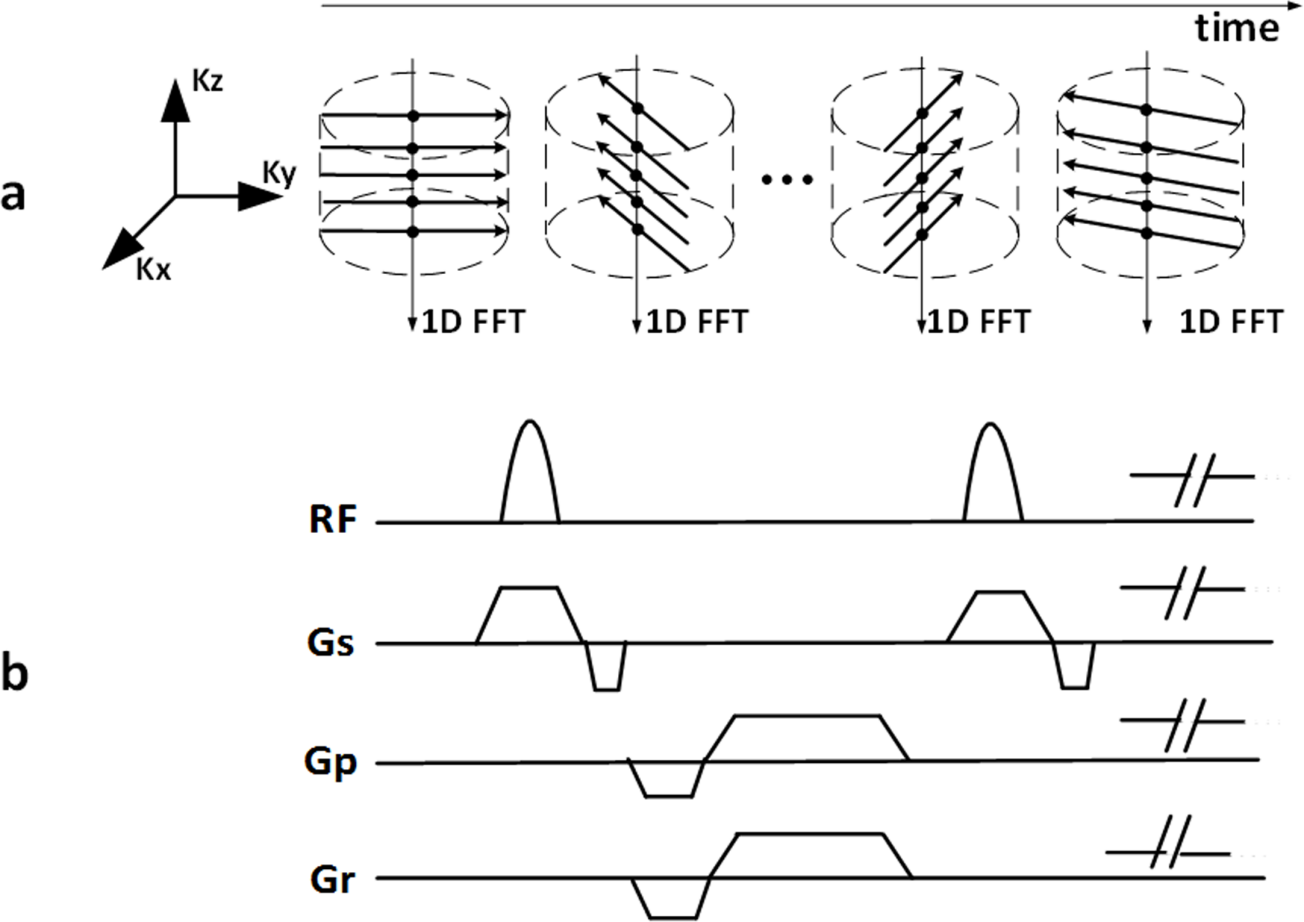
Diagram of the proposed 3D self-gating CMR method for detecting MI in mouse. (a) The stack-of-stars sampling scheme comprises Cartesian encoding along k_z_ direction and radial projections filling in k_x_-k_y_ plane. All partition encodes for a given projection angle are collected sequentially before switching to the next projection angle. This partition-first sampling scheme enables the projection centers (denoted as black solid circles) to be used as self-gating data for extracting cardiac and respiratory motion signals; (b) Corresponding 3D GRE sequence with stack-of-stars sampling trajectories.

Similar to Liu’s method [26], a 3D self-gated GRE sequence with stack-of-stars sampling trajectories was employed for data acquisition, as shown schematically in Fig 1. The stack-of-stars sampling scheme comprises Cartesian encoding along partition (i.e., k_z_) direction and radial projections filling in k_x_-k_y_ plane (Fig 1A). All partition encodes for a given projection angle are collected sequentially before switching to the next projection angle. Such partition-first sampling scheme enables the projection centers (denoted as dots in Fig 1A) to be used as self-gating data for extracting cardiac and respiratory motion signals [26]. Note that the projection angle increases incrementally by 111.246°, which provides approximately uniform k-space with arbitrary number of projection angles.

### MRI protocols

All MR experiments were performed on a 3 T clinical MR system (Tim Trio, Siemens, Erlangen, Germany) without using external ECG trigger and respiratory motion sensor. After a 6-hour reperfusion, a concentration of 1.5 ml/kg body weight gadolinium contrast agent (Consun Pharmaceutical Group Limited, Guangzhou, China) was diluted in 0.5 ml saline and administered via tail vein injection for the enhancement examination. The mouse was then placed head first in the supine position with the heart positioned at the center of a customized four-channel mouse coil and kept warm by a portable wax bag for MR scan. Localizations of mouse cardiac 2-chamber, 4-chamber and short-axis views were done by using a conventional 2D GRE sequence. The sequence parameters for the localization scans included: flip angle = 18°, TR = 8.6 ms, TE = 4.0 ms, field of view = 250 × 250 mm^2^, acquisition matrix = 200 × 256, spatial resolution = 1.3 × 1.0 mm^2^, slice thickness = 4.0 mm, bandwidth = 320 Hz/Pixel. After the localization was completed, the proposed 3D self-gating scan was conducted when gadolinium was injected for approximately 10 minutes to make sure MI area was enhanced. The sequence parameters for the 3D self-gating scan were: flip angle = 18°, TR = 4.2 ms, TE = 2.4 ms, field of view = 58 × 58 × 15 mm^3^, spatial resolution = = 0.3 × 0.3 × 1.5 mm^3^, partition number = 10, bandwidth = 620 Hz/Pixel. Typically, 5–6 consecutive partitions covering the entire heart from the base to the apex. Total 1600 projections were continuously collected for each mouse, corresponding to a scan time of about 1.5 minutes. Note that the spatial resolution of 3D self-gating scan was anisotropic. The spatial resolution along k_z_ direction was 1.5 mm which was far lower than that of the k_x_-k_y_ plane (0.3× 0.3 mm^2^). In order to match the MR images and the histology slices easily, the self-gating scan was localized based on the mouse cardiac 2-chamber, 4-chamber and short-axis views to make the k_x_-k_y_ plane be in coincidence with the short axis view.

### Extracting cardiac and respiratory motion signals

**Fig 2:**
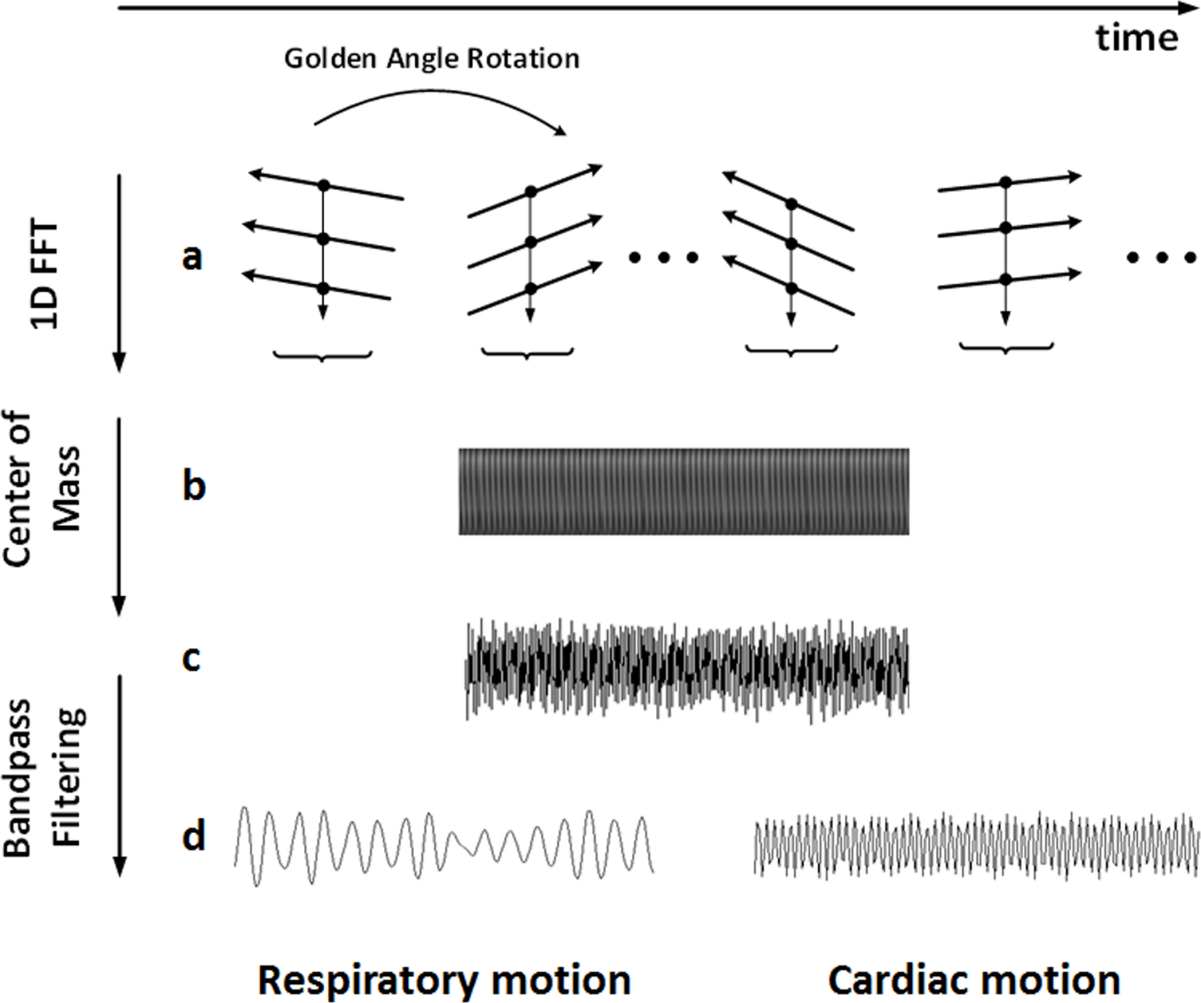
Pipeline of extracting the cardiac and respiratory motion signals. (a) The central points (black solid circles) of each projection angle denote a self-gating data profile. (b) *Z*-intensity profile generated by performing 1D Fourier transform of the self-gating data profiles. (c) Mixed cardiac and respiratory motion signals were obtained by calculating the center of mass from (b). (d) The respiratory and cardiac motion signals extracted by bandpass filtering of (c) and used for data realignment.

Signal processing was conducted offline with MATLAB 2013a (Mathworks Inc., Natick, MA, USA). In order to determine the cardiac and respiratory motion signals, one dimensional Fourier transform was performed on the self-gating data to derive a projection of the entire imaging volume (z intensity profile, Fig 2B). Then the projection translation based on, i.e., center of mass (COM) was analyzed to obtain the mixed cardiac and respiratory motion signals (Fig 2C). Finally, the respiratory and cardiac motion signals was separated by bandpass filtering, which was used to determine the respiratory and cardiac phase for each data segment (Fig 2D) [26]. During the filtering process, the motion signal in the frequency range of 1.0–2.5 Hz was selected to represent respiratory motion, and the motion signal in the frequency range of 6.7–9.2 Hz was selected to represent cardiac motion (based on the mouse respiratory and heart rates: respiratory rate, 60–150 breaths per minute; and heart rate, 400–550 beats per minute). Local minima (valleys) and maxima (peaks) of the extracted respiratory and cardiac motion signals were detected based on these initial rates and were used to iteratively update the rates. In addition, as multiple coil elements were used, an additional virtual coil element, generated by the combination of all real coils using sum of square, was also used for motion detection to provide more motion information. The coil element, including the virtual and real coils, with the smallest variance of respiratory valley position was chosen for respiratory gating; while the coil element with the smallest variance of the detected R—R intervals was selected for cardiac gating. To evaluate the accuracy of the extracting cardiac and respiratory motion signals, the mouse heartbeat and respiratory rates were measured by the same rodent ventilator for 2 min before and after MR scans to obtain the standard heartbeat and respiratory rates for comparison.

### Data realignment

Once the respiratory and cardiac motion signals were obtained, the respiratory and cardiac phases associated with each projection angle were determined. The acquired data were then realigned according to the predetermined respiratory and cardiac phases. Specifically, the acquired data were segmented into six respiratory bins according to the respiratory positions. And then one of the six bins containing the most amounts of projections was adaptively selected for the following cardiac phase segmentation processing. The number of cardiac phases was determined based upon the mean number of projection angles in an R—R interval, and the projections were subsequently assigned to each cardiac phase for image reconstruction. To suppress the effect of arrhythmia, cardiac cycles that were 30% longer or short than the mean number were discarded.

Ideally, the data in cardiac quiescent period can be used for image reconstruction to detect MI. However, it took 42 ms for sampling a self-gating data profile in this study, leading to the self-gating data sampling rates too slow to capture the mouse cardiac quiescent period. Therefore, only the cardiac phase right before each waveform peak (near end-diastole) was used as the quiescent phase, which was similar to the process of previous study [27].

### Image reconstruction

After the data realigned, the projection distribution in each cardiac phase was generally nonuniform and did not satisfy Nyquist-Shannon criteria, resulting in severe streaking artifacts on the reconstructed image using a conventional regridding method. To reduce these artifacts, a CS method derived by exploiting the image temporal sparsity was implemented for image reconstruction (Eq 1):

$$\arg\;\min\left\{\lambda {\left\|T\cdot \rho \right\|}_{1}\right\}\;subject\;to\;{\left\|d-P\cdot F\cdot \rho \right\|}_{2}^{2}<\varepsilon$$ (1)

where *P* is the sampling matrix, *F* is the nonuniform fast Fourier transform operator defined on the radial acquisition pattern, *ρ* denotes the image series to be reconstructed in the x-y-t-coil space, *d* is the acquired radial data in k-t-coil space, *T* represents the temporal total-variation operator imposed on the *l*_1_ norm, and *λ* is the regularization weight that controls the tradeoff between the data consistency and sparsity. A tailored version of the Bregman algorithm was used to solve the optimization problem [28]. Image reconstruction was implemented in the same MATLAB version on a workstation with quad cores and 64 G RAM memory. To demonstrate the advantageous of using proposed CS reconstruction, cardiac images were also reconstructed using a conventional regridding method.

### Histological analysis

After MR scan, all mice were re-anaesthetized and sacrificed by rapid excision of the heart. The heart was then rinsed in 0.9% NaCl, frozen and manually sectioned into 5–6 short-axis slices with thickness of ~1.5 mm from apex to base. The slices were incubated at 37°C with 0.1% triphenyltetrazolium chloride (TTC) for 15 minutes. Both sides of each slice was photographed with a digital camera and prepared for further analysis.

### Image analysis

To assess the accuracy of extracting cardiac and respiratory motion signal, the heart and respiratory rates were calculated from the self-gating signals, and compared with those recorded by the rodent ventilator, averaging before and after MR scans. All data are expressed as mean ± standard deviation.

To demonstrate the advantages of the proposed CS reconstruction, contrast-to-noise ratio (CNR) was calculated based upon previously published methods [14]. Remaining cognizant that noise distribution varies spatially in CS reconstruction, we evaluated the CNR between the left ventricular (LV) myocardial wall and cavity by focusing only on these structures of interest to assess the ability to discriminate wall tissue from the ventricular cavity. Accordingly, the CNR was calculated as Eq 2 [14]:

$$\mathrm{CNR}=\frac{Mean\left(S_{ventricular\;cavity}\right)-Mean\left(S_{myocardial\;wall}\right)}{\sqrt{SD{\left(S_{ventricular\;cavity}\right)}^{2}+SD{\left(S_{myocardial\;wall}\right)}^{2}}}$$ (2)

The CNR of each subject was expressed as the mean ± standard deviation. The mean CNR was statistically analyzed by the Wilcoxon sign-rank test. A *p* value less than 0.05 was considered to be significant.

**Fig 3:**
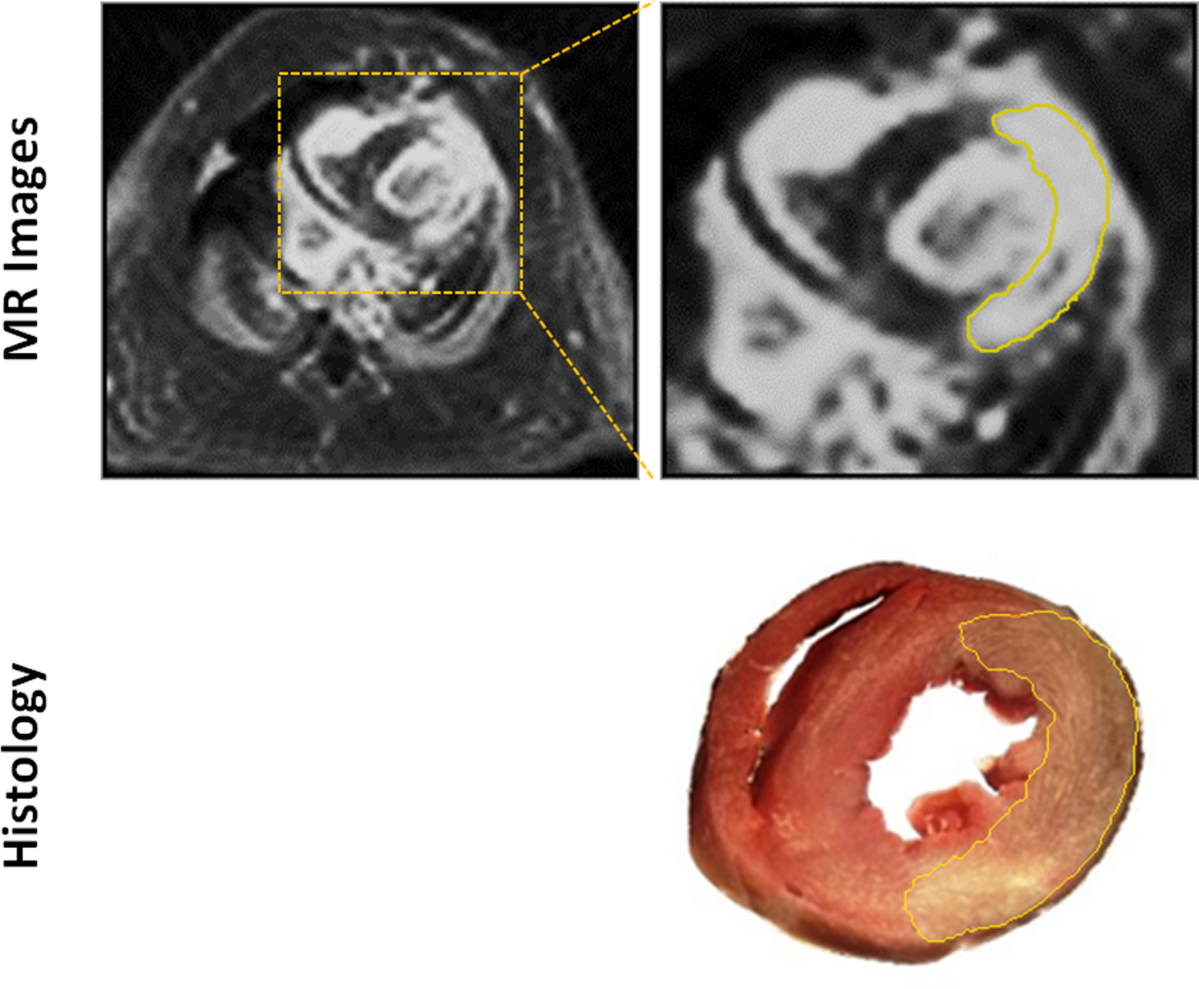
Illustration of contouring the MI areas on both MR and histological images. The boundaries (yellow solid lines) were first determined by Image-Pro Plus software and then manually outlined for reducing the measurement bias.

To evaluate the accuracy of the proposed method for detecting an MI, the areas of MI and myocardium were measured from the matched MR image and the histological picture, respectively. Similar to the method described in [29, 30], the areas of the enhanced (infarct) and non-enhanced (healthy) myocardium in each slice were measured using Image-Pro Plus software (Media Cybernetics, version 6.1). As shown in Fig 3, the boundary of MI was first determined with a local thresholding technique which was automatically performed by Image-Pro Plus software, and then manually outlined for reducing the measurement bias resulting from the similarity between MI and blood pool. Use the same approach to determine the area of left ventricle healthy myocardium, and then the infarct and myocardial area ratio can be calculated by Eq 3.

$$Area_{infarct}/\left(Area_{infarct}+Area_{healthy}\right)$$ (3)

The mean area ratio of each subject was then obtained by averaging the areas of the selected slices in which MIs were identified. Finally, linear regression and Bland-Altman analysis were used to assess the agreement between the proposed method and the histological analysis.

## Results

All MR scans were conducted successfully. The self-gating signals characterizing cardiac and respiratory motion were successfully extracted from all subjects. As summarized in Table 1, the heartbeat and respiratory rates obtained by the proposed self-gating method were well in accordance with those obtained by the rodent ventilator (heartbeat: 430±7 vs. 426±7 bpm; respiratory rate: 78±2 vs. 79±2 bpm).

**Table 1:** Heart and respiratory rates obtained by the proposed self-gating method and the rodent ventilator.

Heart rate (bpm)			Respirtory rate (bpm)	
Mice	Self-gating	Ventilator	Self-gating	Ventilator
**1**	418±5	415±7	84±2	84±2
**2**	433±6	429±4	74±2	78±2
**3**	437±11	431±13	73±1	74±2
**4**	430±10	421±4	76±2	78±2
**5**	439±8	438±12	84±2	81±2
**6**	420±4	420±5	76±1	79±4
**Mean**	430±7	426±±7	78±2	79±2

**Fig 4:**
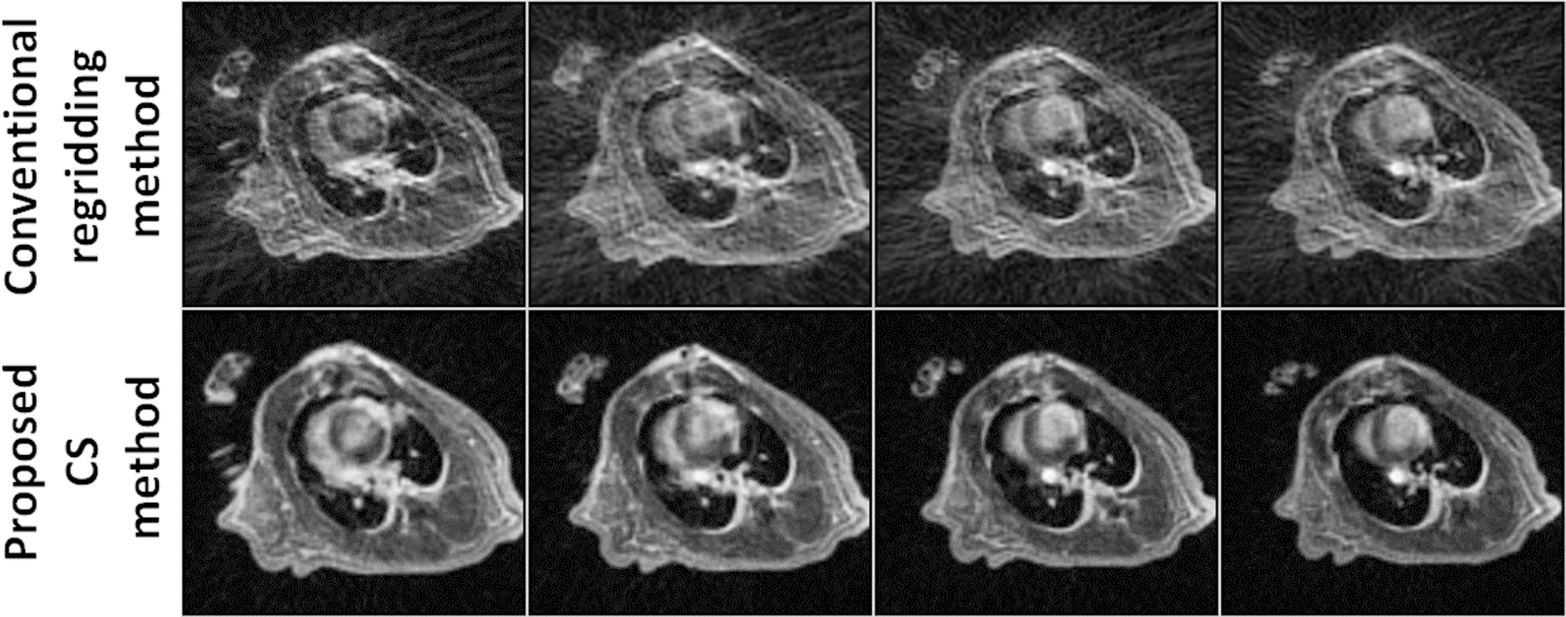
Representative images reconstructed by conventional regridding method and the proposed CS method. Compared with the conventional regridding method, the images reconstructed by the proposed CS method show much less streaking artifacts.

A direct comparison of short-axis views of the left ventricle reconstructed by a conventional self-gated method and the proposed method is shown in Fig 4. Images with much less streaking artifact were obtained using the proposed method. A quantitative assessment in terms of the CNR results for the two reconstruction methods in six subjects is summarized in Table 2. An improved CNR between the blood and myocardium (4.1 ± 2.1 vs. 2.9 ± 1.4, *p* = 0.031) was also realized by the proposed method.

**Table 2:** CNRs between the blood and myocardium for images obtained by conventional regridding method and the proposed CS method.

Mice	1	2	3	4	5	6	Mean
**Coventional** **regridding method**	1.8±1.1	4.9±1.6	2.7±1.7	1.9±1.0	3.2±1.3	2.9±1.6	2.9 ± 1.1
**Proposed** **CS method**	2.5±2.0	7.9±3.1	3.6±1.3	2.8±1.9	4.6±2.1	3.1±2.1	4.1 ±2.1

**Fig 5:**
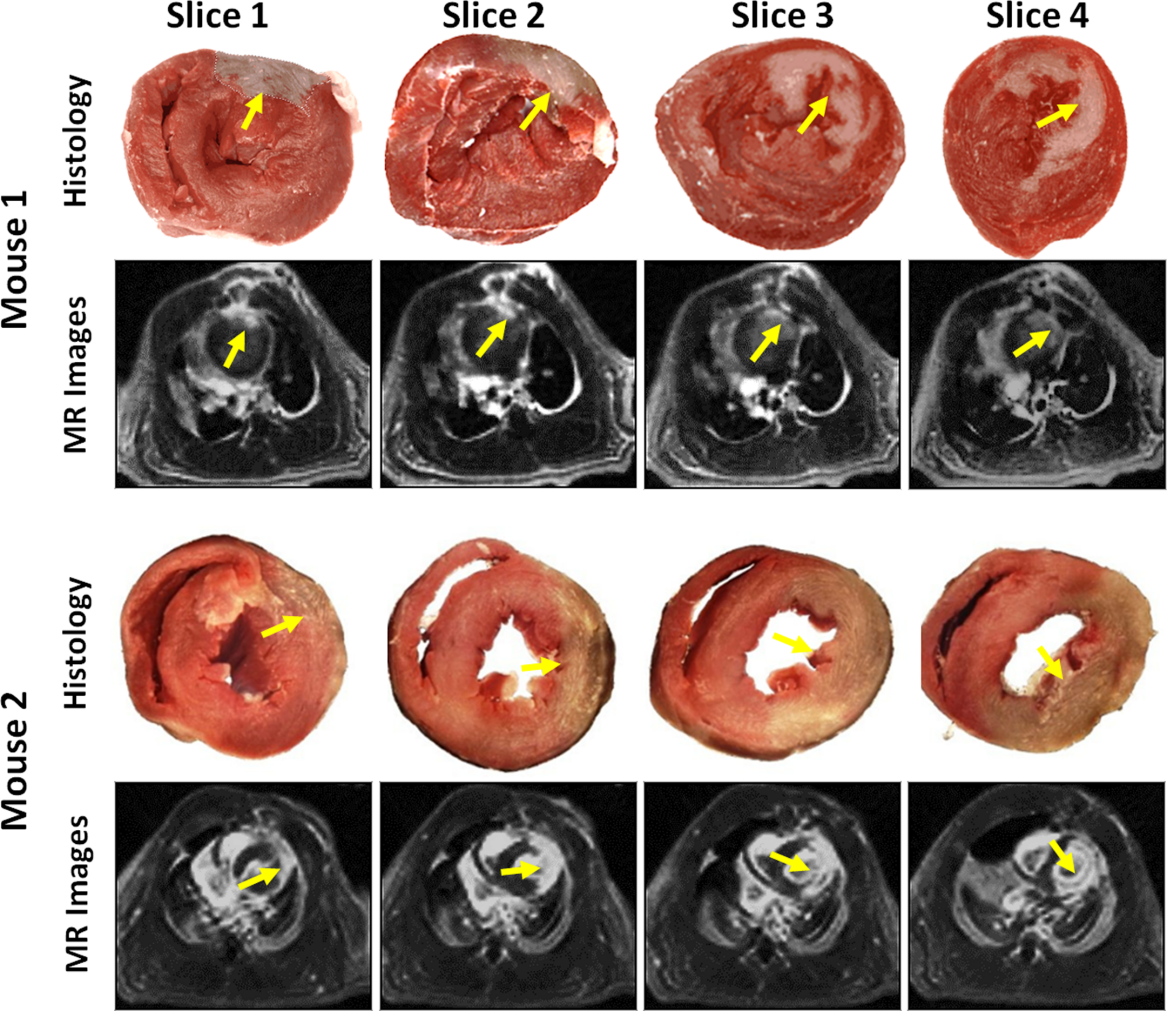
Representative histological pictures and reconstructed MR images from two mice with MI induction. The regions of MI (yellow arrows) matched between the histology analysis and the proposed method.

**Fig 6:**
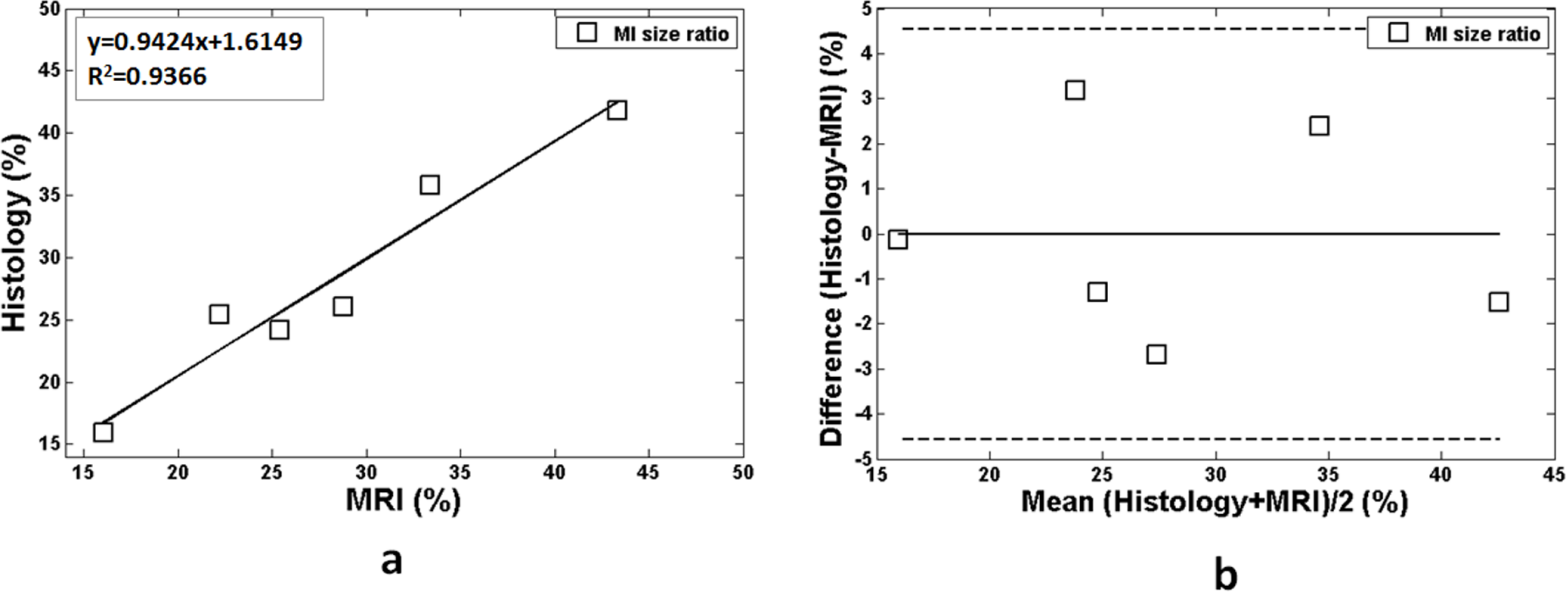
Comparisons of cross-sectional infarction and myocardial size ratio in six subjects obtained with the proposed method and the standard histological analysis approach. (a) Infarction and myocardial area ratio linear regression analysis of the two approaches. (b) Bland-Altman plots of the measurements. Mean difference values are represented by a solid line and the confidence intervals by dashed lines.

The MI regions detected by the proposed method matched those noted by histological analysis (yellow arrows in Fig 5), demonstrating the feasibility of the proposed method for MI detection in mouse. The linear regression analysis showed both good agreement (R^2^ = 0.9366) and a linear relationship (slope = 1.6149) between the proposed method and histological analysis approach (Fig 6A). Additionally, the Bland-Altman plots suggested good performance with narrow limits of agreements between the two approaches (Fig 6B).

## Discussion

In this study, we developed a 3D self-gated imaging method that employs a T1-weighted GRE sequence with stack-of-stars sampling trajectories to detect MI in a mouse model at a 3T clinical MR system. Our preliminary *in vivo* study demonstrated that MIs detected by the proposed method was also match well with those by histological analysis.

There are several important aspects that assure the success of the proposed method for detecting MI in the mouse. First, a large flip angle of 18^o^ was used in the GRE sequence to facilitate heavy T1-weighted imaging. It has previously been reported that post-contrast, T1-weighted imaging techniques are sensitive for the detection and delineation of MI [13]. Using the heavy T1-weighted sequence and gadolinium-based contrast agents, it is possible to accurately define MI as it exhibits delayed hyperenhancement due to a loss of cell and tissue integrity associated with an increase in extracellular volume. Second, the use of stack-of-stars sampling trajectories in the GRE sequence is beneficial for 3D self-gated cardiac imaging without the need of acquiring additional self-gating data. This is because Cartesian mapping of the k-space center along partition direction provides intensity-weighted position information, from which both respiratory and cardiac motion can be derived and thus used for data realignment [26]. Third, the radial sampling trajectory in-plane (k_x_-k_y_) is robust against motion artifact. A previous study described the finding that radial sampling is less susceptible to motion due to its lower sensitivity to motion-induced phase shifts and signal averaging at the center of the k-space [31]. Fourth, the data were empirically segmented into six respiratory bins to reduce respiratory motion artifacts. It is understood that the larger the number of respiratory bins, the better the elimination of respiratory motion artifacts. However, as the number of respiratory bins increases, many more radial projections are needed for image reconstruction, resulting in a longer MR scan for data acquisition. To effectively eliminate the respiratory artifacts while maintaining a reasonable scan time, six respiratory states were empirically chosen for image reconstruction based on previously studies [32, 33].Fifth, anisotropic, rather than isotropic, spatial resolution of the 3D self-gating scan was conducted for correctly extracting the cardiac motion signal. This is because the mouse heartbeat is too rapid. If we want to improve the spatial resolution along partition direction, the number of partitions has to be increased to cover the mouse whole heart. As the number of partitions increased, the temporal resolution between successive projection angles become lower, resulting in the self-gating data sampling rate too low to extracting the cardiac motion signal. Thus, the spatial resolution along the partition direction was set as 1.5 mm, which is lower than that in k_x_-k_y_ plane. Nevertheless, our experiments demonstrated that the detection of MI using the anisotropic scan was well agreement with the histological analysis.

Compared with the conventional 3D self-gated method employed in the mouse [22, 23], the use of CS reconstruction in the proposed method is beneficial for the studies involving MI. First, CS reconstruction can accurately rebuild an image from a sparse sampling dataset and thus reduce MR scan time. In this study, only 1600 radial projections (corresponding to a ~1.5 minute MR scan) were used for image reconstruction. The data assigned to each cardiac phase included 45–86 radial projections, a goal that was not met using the Nyquist-Shannon sampling criteria. However, CS reconstruction by exploiting the image temporal sparsity had much less streaking artifacts compared with the conventional regridding method and did not compromise the detectability of small anatomical heart structures (Figs 4 and 5). Second, CS reconstruction can improve the image CNR under conditions of sparse data sampling. This is because the performance of the conventional regridding method deteriorates significantly if the k-space values are azimuthally undersampled, leading to potential streaking artifacts in the reconstructed images [34].

The self-gated method presented here was performed at 3T clinical MR system, which may be valuable for such an animal study. Due to the small size of the mouse, imaging of mouse cardiac MI is typically performed at high-field (≥ 4.7 T) animal MR system for achieving high spatial resolution images [13]. However, the availability of such systems is limited by their high costs, which may hinder clinical translation. Although CMR imaging in mice has been successfully performed on clinical systems at 1.5–3 T [35], only a few attempts have been made to evaluate MI in mouse as part of a clinical system [36–38]. Here, the proposed technique would be a supplementary alternative strategy at such kinds of clinical system and would be clinically feasible for the evaluation of MI in mice.

This study had two limitations. First, off-line reconstruction remains a problem for routine application. Although the stack-of-stars sampling pattern helps to enable parallelized slice-by-slice reconstruction, the CS reconstruction remains time-consuming in its current version. This issue may be addressed using parallel GPU [39, 40]. Second, current study did not use rigorous mathematical criteria to select the regularization parameter for image reconstruction. The difficult decision on how to select the optimal regularization parameters is not unique to the proposed algorithm but is a general problem for most regularization reconstruction methods [41].

## Conclusion

In conclusion, a 3D self-gated cardiac imaging technique, using a T1-weighted stack-of-stars GRE sequence and CS, was developed for the evaluation of MI in mouse model. A preliminary *in vivo* study demonstrated that the proposed approach can accurately detect MIs in mice in 3T clinical MR systems without the use of external ECG and respiratory motion sensor.
